# Hydrocephalus in prematurity: does valve choice make a difference?

**DOI:** 10.1007/s00381-023-06204-8

**Published:** 2023-11-07

**Authors:** Benjamin J. Hall, Ahmad M.S. Ali, Dawn Hennigan, Benedetta Pettorini

**Affiliations:** 1 Department of Neurosurgery, Alder Hey Children’s Hospital NHS Foundation Trust, Liverpool, UK; 2 Department of Neurosurgery, The Walton Centre NHS Foundation Trust, Liverpool, UK; 3 Institute of Infection, Veterinary and Ecological Sciences (IVES), The University of Liverpool, Liverpool, UK

**Keywords:** Prematurity, Intraventricular haemorrhage, Hydrocephalus, Programmable, Valve

## Abstract

**Purpose:**

Extremely premature neonates diagnosed with post-haemorrhagic hydrocephalus (PHH) are recognised to have particularly poor outcomes. This study assessed the impact of a number of variables on outcomes in this cohort, in particular the choice of shunt valve mechanism.

**Methods:**

Electronic case notes were retrospectively reviewed of all premature neonates admitted to our centre for management of hydrocephalus between 2012 and 2021. Data included (i) gestational age, (ii) birth weight, (iii) hydrocephalus aetiology, (iv) surgical intervention, (v) shunt system, (vi) ‘surgical burden’ and (vii) wound failure and infection rate. Data was handled in Microsoft Excel and statistical analysis performed in SPSS v27.0

**Results:**

*N* = 53 premature hydrocephalic patients were identified (*n* = 28 (52.8%) female). Median gestational age at birth was 27 weeks (range: 23–36 + 6 weeks), with *n* = 35 extremely preterm patients and median birth weight of 1.9 kg (range: 0.8–3.6 kg). Total *n* = 99 programmable valves were implanted (*n* = 28 (28.3%) de novo, *n* = 71 (71.2%) revisions); *n* = 28 (28.3%) underwent *n* ≥ 1 pressure alterations, after which *n* = 21 (75%) patients had symptoms improve. In *n* = 8 patients exchanged from fixed to programmable valves, a mean reduction of 1.9 revisions per patient after exchange was observed (95%CI: 0.36–3.39, *p* = 0.02). Mean overall shunt survival was 39.5 weeks (95%CI: 30.6–48.5); 33.2 weeks (95%CI: 25.2–41.1) in programmable valves and 35.1 weeks (95%CI: 19.5–50.6) in fixed pressure (*p* = 0.22) with 12-month survival rates of 25.7% and 24.7%, respectively (*p* = 0.22). Shorter de novo shunt survival was associated with higher operation count overall (Pearson’s *R*: − 0.54, 95%CI: − 0.72 to − 0.29, *p* < 0.01). Wound failure, gestational age and birth weight were significantly associated with shorter de novo shunt survival in a Cox regression proportional hazards model; gestational age had the greatest impact on shunt survival (Exp(B): 0.71, 95%CI: 0.63–0.81, *p* < 0.01).

**Conclusion:**

Hydrocephalus is especially challenging in extreme prematurity, with a shorter de novo shunt survival associated with higher number of future revisions. Programmable valves provide flexibility with regard to pressure setting, with the potential for fewer shunt revisions in this complex cohort.

## Introduction

The impact of prematurity on neurodevelopment is manifold, with germinal matrix–intraventricular haemorrhage (IVH) being the most commonly diagnosed brain lesion in preterm infants. Despite improvements in perinatal care leading to an apparent reduction in incidence [1], between 15 and 20% of infants weighing less than 1500 g at birth will develop IVH [2, 3], with a subgroup developing post-haemorrhagic hydrocephalus (PHH) that requires surgical intervention. Historically, early implantation of definitive CSF diversion measures such as ventriculoperitoneal (VP) or ventriculoatrial (VA) shunts in PHH has been associated with poor outcomes, with high rates of infection and shunt failure [4]. Thus, a catalogue of temporising measures has been introduced to postpone the need for definitive shunting, ranging from serial lumbar punctures and ventricular taps through to ventriculosubgaleal shunts (VSGS) and neuro-endoscopic lavage (NEL) [5]. Each of these treatment modalities is accompanied by varying risks and benefits and is often employed according to practitioner choice. Whilst the debate surrounding the optimal temporising modality continues, they often remain as temporising measures alone, with a number of the more severe IVH patients going on to require definitive shunt implantation. This subgroup of shunted hydrocephalus patients has been reported as having poorer neurodevelopmental outcomes [6], as well as substantial morbidity when it comes to shunt-related surgeries, with increased rates of infection [4], revision rates of between 4 and 6.9 shunts per patient [4, 7], all compounded by an increased risk of developing multiloculated hydrocephalus [8, 9]. Thus, optimising patients with PHH in every way possible to minimise the need for recurrent surgical intervention is of the utmost importance. Shunt valve choice remains a controversial topic across neurosurgery and is not limited to paediatrics. A paucity of randomised trial data has hindered objective comparison between valve designs, with recent evidence suggesting no single design more effective than any other in an infant population [10]. The particular complexities associated with the premature neonate population, including their increased risk of slit ventricle syndrome, loculated hydrocephalus and infection, necessitate a more thorough review of shunt valve utilisation. This study therefore reviewed a single centre’s experience over 10 years of managing hydrocephalus in a premature neonatal population, in doing so describing risk factors associated with particularly poor outcomes.

## Methods

A retrospective review of all premature neonates admitted to our paediatric neurosurgical department between 2012 and 2021 for CSF diversion was performed. Electronic case notes were reviewed for patient details including (i) gestational age at birth, (ii) birth weight, (iii) aetiology of hydrocephalus, (iv) grade of IVH and (v) need for and modality of temporising CSF diversion. Degree of prematurity was stratified into the subgroups (i) extremely premature (< 28 weeks), (ii) very premature (28–31 + 6 weeks), (iii) moderately premature (32–33 + 6 weeks) and (iv) late premature (34–36 + 6 weeks). Birth weight was stratified into the subgroups (i) extremely low (< 1 kg), (ii) very low (1–1.49 kg), (iii) low (1.5–2.5 kg) and (iv) normal (> 2.5 kg). Outcome data collection included (i) need for definitive CSF diversion; (ii) infection and wound failure rates; (iii) valve choice; (iv) overall shunt survival; (v) ‘surgical burden’, defined as the number of hydrocephalus-related operations undertaken during follow-up and (vi) mortality. All cases were diagnosed due to either increasing ventricular indices on cranial ultrasound or following investigations for increasing head circumference. Of note, the treatment protocol for CSF diversion at our centre mandates that patients be above 2 kg in weight prior to any definitive shunt implantation.

Data handling was performed in Microsoft Excel and statistical analysis in SPSS v27.0. Overall shunt survival is illustrated using Kaplan–Meier curves with log-rank testing to assess for statistically significant differences. To assess relative impact on shunt survival, variables demonstrating a *p*-value of ≤ 0.2 on log-rank testing were included for further analysis in a Cox regression proportional hazards model. Comparison of dichotomous variables is presented descriptively and compared using the Chi-squared test, ordinal data was assessed using the Mann–Whitney U test and means were compared using the independent samples *t*-test. Ranges (*R*), 95% confidence intervals (95%CI:) and standard deviations (SD) are reported where appropriate, and statistical significance threshold was set at *p* < 0.05.

## Results

### Patient cohort

*N* = 53 premature patients were admitted for CSF diversion during the interval reviewed, of whom *n* = 28 (52.8%) were female. Median gestational age at birth was 27 weeks (range: 23–36 + 6 weeks), with *n* = 35 extremely preterm, *n* = 11 very preterm, *n* = 2 moderate preterm and *n* = 2 late preterm patients. Median birth weight was 1.9 kg (range: 0.8–3.6 kg); *n* = 3 patients had extremely low, *n* = 10 very low, *n* = 21 low and *n* = 11 patients had normal birth weights. Aetiology of hydrocephalus was idiopathic in *n* = 1 patient, infection in *n* = 3 patients and IVH in *n* = 49 patients, of which *n* = 2 were Grade 2, *n* = 8 were Grade 3 and *n* = 39 were Grade 4.

### Surgical intervention

Temporising measures for early hydrocephalus included NEL in *n* = 8 (15.0%) patients, whilst *n* = 41 (77.4%) received an VSGS, *n* = 2 (3.8%) underwent EVD insertion and *n* = 2 (3.8%) underwent Ommaya reservoir insertion. Wound issues following temporisation were encountered in *n* = 13 (24.5%) patients, in whom *n* = 8 (15.0%) developed wound leaks alone, whilst *n* = 5 (9.4%) also developed CNS infections following leak. Overall, *n* = 45 (84.9%) patients required definitive CSF diversion in the form of a ventricular shunt, whilst *n* = 4 (7.5%) patients did not require definitive diversion at all. *N* = 3 (5.7%) patients died in the perinatal period, and *n* = 1 (1.9%) patient was transferred to their local unit within a few days of birth and was therefore lost to follow-up. Neither birth weight (*X*^2^: 11.2, *p* = 0.26) nor degree of prematurity (*X*^2^ = 6.5, *p* = 0.69) was significantly associated with choice of temporising measure. Within the shunted cohort, the first shunt was VP in *n* = 40 (88.9%) and VA in *n* = 5 (11.1%); *n* = 3 (6.7%) patients with VP shunts were converted to VA during follow-up and vice versa in *n* = 1 (2.2%). Median uncorrected age at implantation of first shunt was 12.9 weeks (range: 2.1–40.6).

### Shunt valve data

**Fig. 1:**
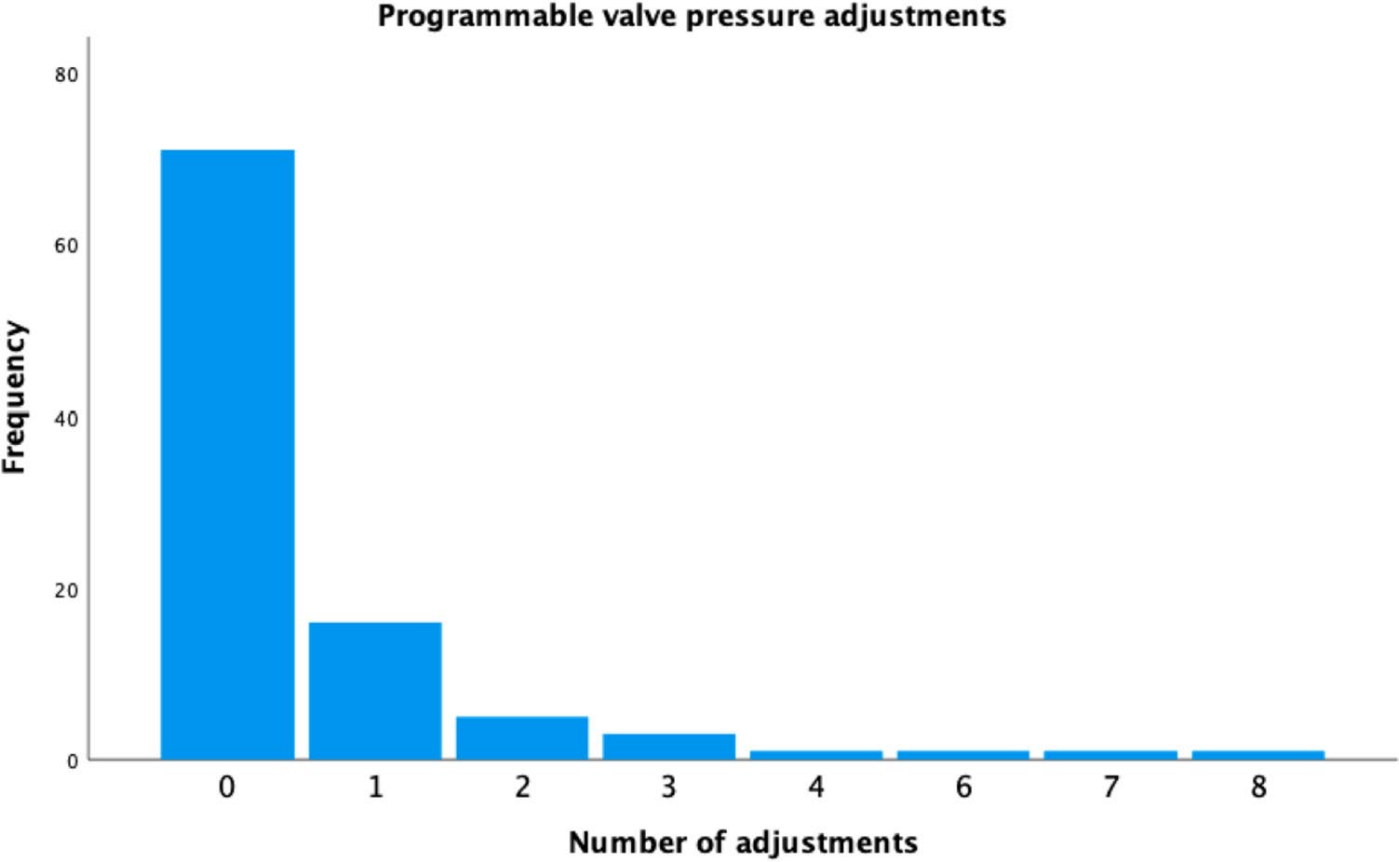
Total programmable valve pressure adjustments per patient, undertaken during follow-up

One hundred thirty-nine valves were implanted in this population; *n* = 45 de novo and *n* = 94 revisions. In the de novo setting, *n* = 28 (62.2%) programmable valves were used and *n* = 17 (37.8%) fixed pressure, whilst overall there were *n* = 99 programmable valves implanted and *n* = 40 fixed pressure. Fixed pressure models included *n* = 28 (20.1%) Miethke 4/24 and *n* = 12 (8.6%) were Miethke 9/29; within the programmable cohort *n* = 35 (25.2%) proGAV2.0, *n* = 7 (5.0%) MBlue plus, *n* = 56 (40.3%) Codman MicroHakim and *n* = 1 (0.7%) Codman Certas. *N* = 8 (47.1%) patients who had fixed pressure valves implanted de novo had them revised to programmable valves during follow-up. No patients receiving de novo programmable valves were swapped to fixed pressure, though in *n* = 4 (14.2%) patients with programmable valves and an anti-gravity unit were switched to a programmable differential pressure valve alone in order to achieve an overall lower drainage pressure. Neither birth weight (*X*^2^ = 3.6, *p* = 0.31) nor degree of prematurity (*X*^2^ = 3.9, *p* = 0.28) was significantly associated with choice of first valve. Shunt revision was due to proximal blockage in *n* = 60 (63.8%) cases, though whether catheter or valve block was specifically responsible was frequently not specified. Other causes of failure were infection in *n* = 15 (16.0%) cases, disconnection of proximal catheter in *n* = 2 (2.1%) cases and abdominal failure in *n* = 5 cases (5.3%). Revision was performed semi-electively in order to lengthen a distal atrial catheter in *n* = 4 (4.3%) cases and in *n* = 4 (4.3%) cases to exchange to programmable valves without an anti-gravity mechanism in order to achieve a lower pressure. Failure of the valve mechanism was specified as indication for revision in *n* = 4 (4.3%) cases; *n* = 3 of which were Codman MicroHakim and *n* = 1 was a Miethke ProGAV2.0. Of the programmable valves implanted, *n* = 71 (71.7%) did not have their pressure setting adjusted, *n* = 16 (16.2%) patients underwent 1 pressure adjustment and *n* = 12 (12.1%) underwent 2 or more (Fig. 1). Alteration of valve pressure settings improved symptoms in *n* = 21 (75%) of cases. The mean number of operations per patient was slightly lower in those receiving programmable (*n* = 5.2, SD: 2.8) valves as part of their de novo shunt system as opposed to fixed pressure (*n* = 6.1, SD: 4.0), though this was not statistically significant (*p* = 0.10). In the group that were swapped from fixed to programmable valves, the mean number of revisions per patient prior to implantation of a programmable valve was *n* = 2.88, compared to *n* = 1 postop, thus a significant mean reduction of 1.9 revisions per patients (95%CI: 0.36–3.39, *p* = 0.02).

### Outcome data

**Fig. 2:**
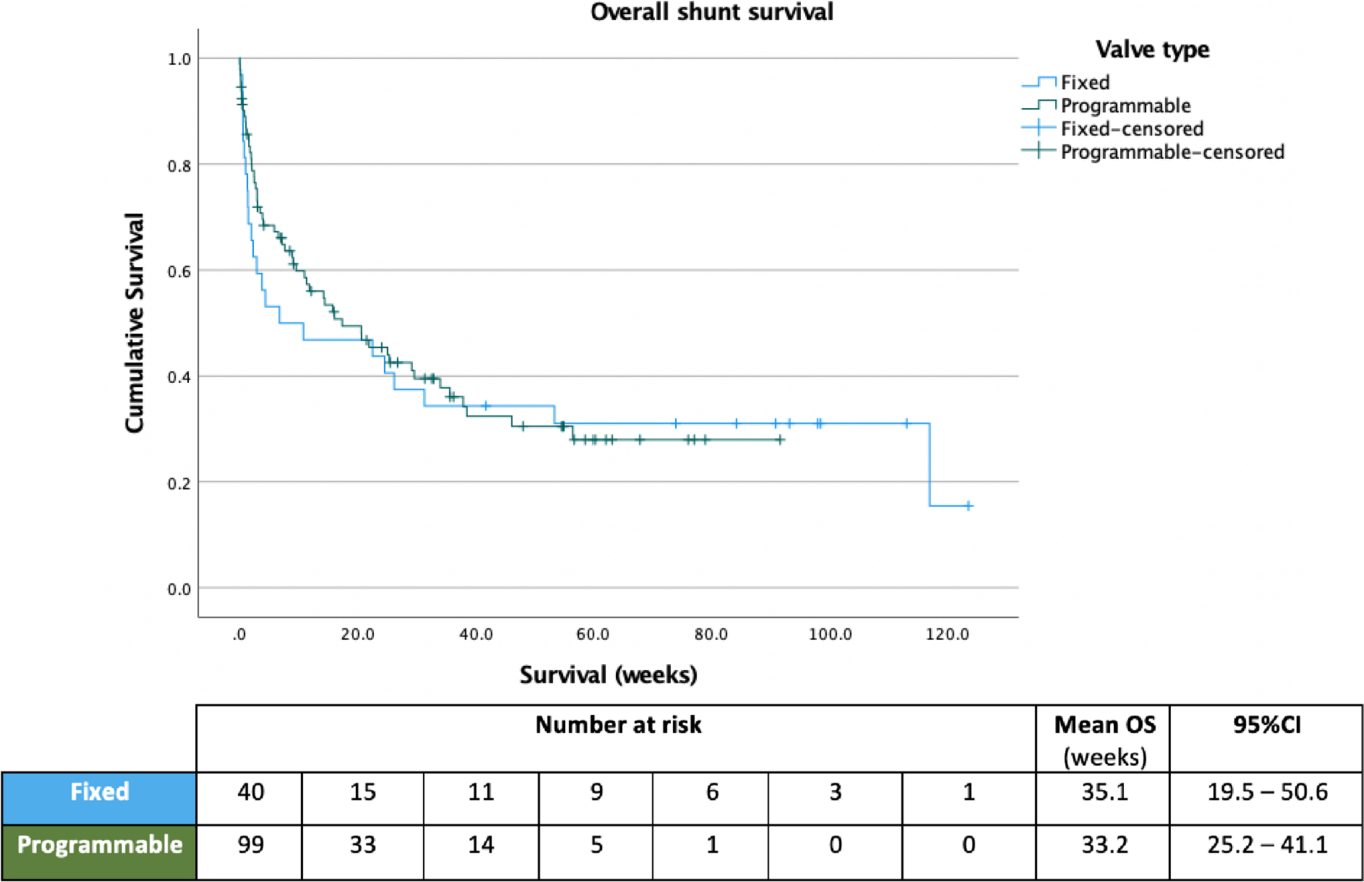
Overall shunt survival and number at risk, stratified according to valve type

Mean overall shunt survival was 39.5 weeks (95%CI: 30.6–48.5); excluding revisions due to infection this was 44.3 weeks (95%CI: 34.5–54.0). Mean survival for de novo shunts was 44.8 week (95%CI: 27.4–62.2) compared to 44.1 weeks (95%CI: 32.3–56.0) in revisions (*p* = 0.88). Mean valve survival was 33.2 weeks (95%CI: 25.2–41.1) in programmable valves and 35.1 weeks (95%CI: 19.5–50.6) in fixed pressure (*p* = 0.22) (Fig. 2), with 12-month survival rates of 25.7% and 24.7%, respectively (*p* = 0.22).

**Fig. 3:**
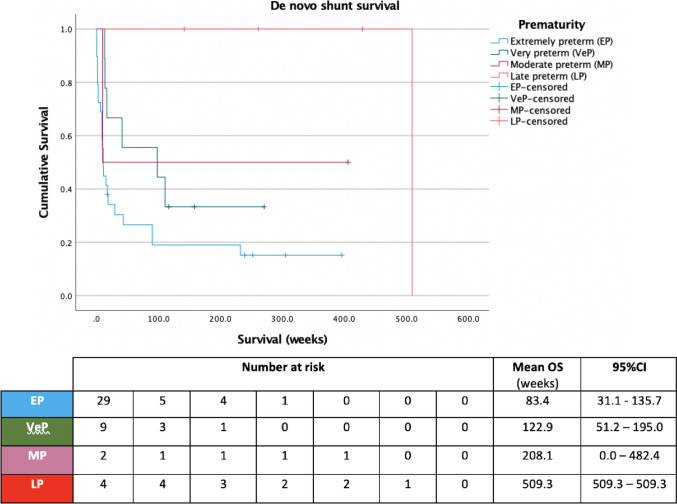
Shunt survival stratified according to degree of prematurity

**Fig. 4:**
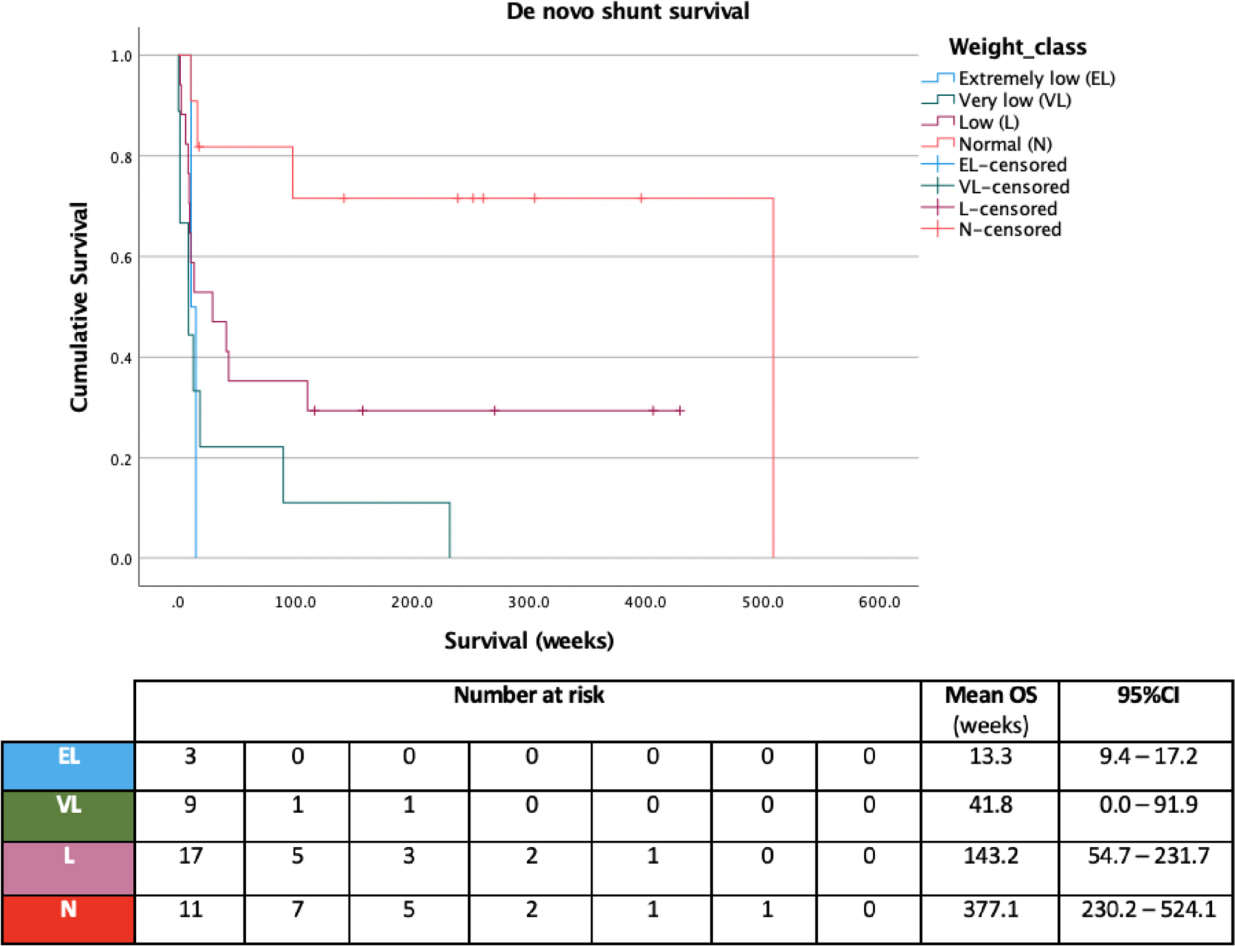
Shunt survival stratified according to birth weight

Gestational age was found to have a strong positive correlation with de novo shunt survival (Pearson’s *R*: 0.62, 95%CI: 0.39–0.77, *p* < 0.01), also demonstrated when stratified into groups according to degree of prematurity (*p* = 0.02, Fig. 3). Gestational age also demonstrated a moderate negative correlation with overall number of operations (Pearson’s *R*: − 0.42, 95%CI: − 0.62 to − 0.17, *p* < 0.01). Birth weight was moderately positively correlated with de novo shunt survival (Pearson’s *R*: 0.41, 95%CI: 0.13–0.63, *p* < 0.01), including when stratified according to weight class (*p* < 0.01, Fig. 4), but was not correlated with number of operations (Pearson’s *R*: − 0.11, 95%CI: − 0.37 to − 0.17, *p* = 0.44). Aetiology of hydrocephalus, modality of temporising measure and choice of valve mechanism were not significantly associated with de novo shunt survival (*p* > 0.2).

**Fig. 5:**
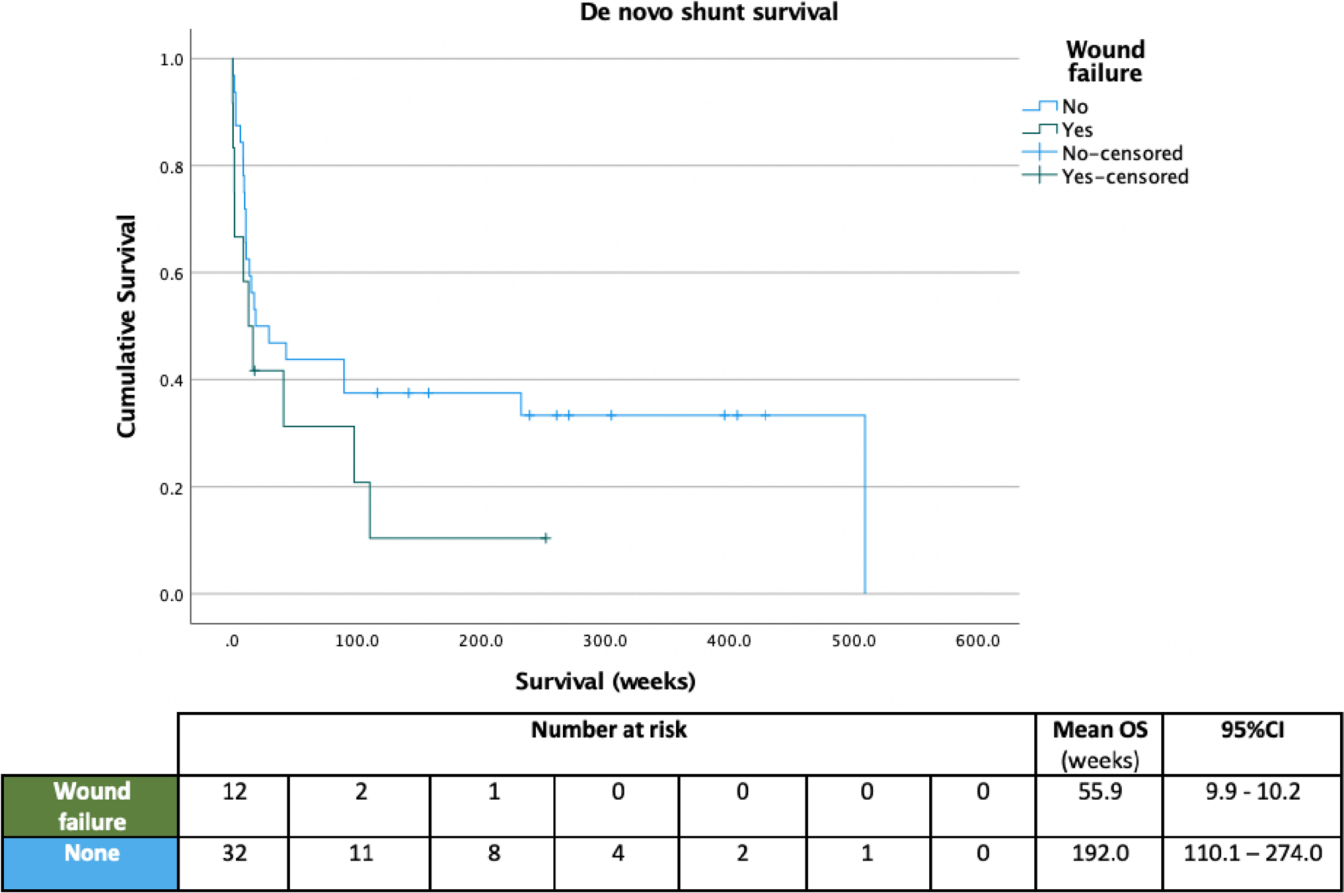
Overall shunt survival and number at risk, stratified according to the presence of wound issues following initial temporising surgery for hydrocephalus

Patients encountering wound issues related to their initial temporising intervention had a substantially shorter mean survival of their first shunt of 55.9 weeks (95%CI: 9.9–102.0) compared to 192.0 weeks in those who did not (95%CI: 110.1–274.0), though this difference was not statistically significant (*p* = 0.16, Fig. 5). The likelihood of encountering a wound issue was not significantly related to either gestational age or birth weight (*p* = 0.9 and 0.09, respectively). The overall survival time of de novo shunts was significantly, negatively associated with overall number of operations (Pearson’s *R*: − 0.54, 95%CI: − 0.72 to − 0.29, *p* < 0.01). Variables found to have a *p*-value of ≤ 0.2 on log-rank testing in relation to de novo shunt survival were ‘wound failure after initial surgery’, gestational age and birth weight. A statistically significant Cox regression proportional hazards model was created with inclusion of all three variables and gestational age as having the greatest impact on shunt survival (Exp(B): 0.71, 95%CI: 0.63–0.81, *p* < 0.01) (Table 1).

**Table 1:** Cox regression proportional hazards model (significance *p* < 0.05)

**Variable**	**Exp(B)**	**95%CI**	***p*-value**
**Gestational age**	0.75	0.63–0.89	0.001
**Birth weight**	0.58	0.42–0.79	0.001
**Wound failure**	0.28	0.11–0.68	0.005

## Discussion

The complexities of managing younger patients with hydrocephalus have long been recognised, with prematurity only serving to exacerbate the risks further [11]. This study emphasises the impact of both low birth weight and extreme prematurity on shunt survival, with both resulting in significantly shorter de novo shunt survival. This cohort represents the more severe subgroup of those with PHH, given that all were transferred for temporising measures in the neonatal setting. Whilst a majority of patients presented with Grade 4 IVH, the inclusion of ten milder cases of IVH highlights the importance of close ventricular index and head circumference monitoring during the neonatal period to ensure all PHH is identified as and when it arises.

Whilst 24-month survival was not reportable due to limited follow-up, 12-month survival rates in this cohort are dramatically shorter than those from our own infant cohort [10], highlighting the complexity of this patient group. Of note, time to failure of de novo shunts in this series was negatively correlated with total number of operations (Pearson’s *R*: − 0.54, *p* < 0.01), implying that the longer the initial shunt survival, the fewer total re-interventions were required during the patient’s childhood. Thus, this supports the notion that optimising the first shunt implantation is of the utmost importance.

Whilst birth weight and gestational age are not necessarily modifiable risk factors, this study demonstrates that high-quality wound care is. Whilst this variable was not significantly associated with shunt survival in univariate analysis, likely due to the small sample size, it did become significant when included in a multivariate model (*p* < 0.01, Table 1). The functional immaturity of neonatal skin is exacerbated in prematurity, particularly in those less than 24 weeks [12], leading to the potential for wound breakdown when exogenous materials such as shunt catheters and valves are implanted. Those experiencing wound issues following their temporising interventions in this cohort were demonstrated to have a shorter de novo shunt survival; therefore, careful wound monitoring must be guaranteed during their postoperative recovery. Nutritional optimisation is also critical to healthy wound healing; therefore, early dietetic involvement to support patients and their caregivers is key. Given that patients with VSGS may be discharged home or transferred to non-neurosurgical centres whilst awaiting definitive shunt implantation, it is also critical that suitable wound care counselling for parents and caregivers is provided prior to discharge, in order to recognise any concerns early on.

Whilst our centre previously reported little evidence to suggest superiority between valve types in infants (10), in this particularly complex premature cohort, it is a variable that should be considered carefully. Again, neither overall nor de novo shunt survival was significantly different between fixed and programmable valve groups (*p* = 0.73); however, when considering total number of operations, there were fewer per patient in those initially receiving programmable valves compared to those with fixed pressure (5.2 vs 6.1, respectively, *p* = 0.10). Whilst this was not a statistically significant discrepancy, the mean reduction of 1.9 revisions per patient (95%CI: 0.36–3.39, *p* = 0.02) following implantation of a programmable valve in those that initially received fixed pressure valves demonstrates that patient selection likely plays a significant role. Similarly, a number of patients with programmable valves that did require revision were those in whom the pressure required was lower than that obtainable with an anti-gravity component in situ. These caveats suggest that rather than any single superior valve model, it is patient selection that must be improved. Pre-emptively identifying high-risk patients such as those at risk of low-pressure hydrocephalus is notoriously challenging; therefore, analysis of larger cohorts is required to provide guidance in this area and further limit the number of repeat shunt surgeries in this population.

Shunt survival itself remains the pre-eminent metric when evaluating outcomes in hydrocephalus, but the acceptability of this is debated as increasing attention has been paid to neuro-cognitive outcomes in hydrocephalus, as evidenced by DRIFT and IIHS [13]. The susceptibility to brain injury of premature neonates makes utilising such outcome measures particularly important and should certainly be considered in future studies. Whilst neuro-cognitive outcomes were beyond the scope of this study, surgical morbidity and the need for recurrent shunt operations were evaluated. The impact of multiple surgeries, specifically on cognitive development, remains to be seen, but the risks presented with each general anaesthetic as well as the cost of each operation are in and of themselves, poor outcomes.

Parents and caregivers play an instrumental role in ensuring the best positive outcomes for these patients, through close monitoring at home and early recognition of shunt malfunction. Little research has objectively assessed the impact of such profound clinical responsibilities on the families of patients, though it has been reported to lead to depression and anxiety in around a fifth [14]. A multidisciplinary approach to supporting these patients is crucial to ensure good outcomes, with early involvement of psychology teams to ensure families are sufficiently supported.

## Limitations

As discussed, lack of neuro-cognitive outcome data remains a shortcoming of much hydrocephalus research, and this study is no exception. Future work will look to include prolonged follow-up and assessments of cognition alongside this. Shunt malfunctions are often multifactorial, with ‘proximal blockages’ often occurring as a result of either or both ventricular catheter and valve obstruction. As such, details regarding proximal revisions and the components revised were not always available. If the valve mechanism had failed this was specified, but often in proximal blocks, there is empirical revision of both proximal catheter and valve. Consistency in operation notes and inclusion of such detail in future will provide a more thorough analysis of valve efficacy.

Importantly, this series specifically observes those patients at the most severe end of the PHH spectrum. Whilst extremely low birth weight and extreme prematurity are evidently predictors of poor outcome during childhood, comparison with other age groups and their long-term outcomes into adolescence and adulthood is an important goal for future work.

## Conclusion

Extremely premature patients are known to have an increased propensity for developing IVH, and this study demonstrates that the course of their resultant hydrocephalus is often more complex than those born nearer term. Early wound failure, lower gestational age and birth weight are predictors of shorter de novo shunt survival, which in turn is correlated with higher surgical burden in future. Whilst overall survival is not significantly different between programmable and fixed pressure shunt valves, the former appear to reduce the number of revisions per patient in certain cases and therefore retain an important role in the care of this complex patient cohort.

## Data Availability

The datasets generated and/or analysed during the current study are available from the corresponding author on reasonable request.
